# Beyond the Appendix: Unveiling Florid Lymphoid Hyperplasia

**DOI:** 10.7759/cureus.103103

**Published:** 2026-02-06

**Authors:** Apoorva Raichur, Girish Bakhshi

**Affiliations:** 1 General Surgery, Grant Government Medical College and Research Institute, Mumbai, IND

**Keywords:** appendicitis, appendix, florid, hyperplasia, lymphoid

## Abstract

Florid lymphoid hyperplasia (FLH) is a rare entity with an unknown etiology. It may occur anywhere in the gastrointestinal tract, from the base of the tongue to the anus, but is more common at the terminal ileum and rectum. It often presents as an acute abdominal condition. Our case illustrates how FLH can closely mimic the clinical presentation of acute appendicitis. The resemblance can be so striking that it poses a significant diagnostic challenge, highlighting the importance of careful evaluation in such scenarios.

## Introduction

Florid lymphoid hyperplasia (FLH) is an uncommon condition with an unclear cause. It was initially documented by Briquet in 1838, and later, in 1941, Marina-Fiol and Rof-Carballo explored it through radiological methods and renamed it “enteritis follicularis” [1].

Five instances involving the ileocecal region have been documented [2]. Although the disease may occur anywhere in the gastrointestinal tract, from the base of the tongue to the anus, it is more common at the terminal ileum and rectum. It often presents as an acute abdominal condition. It is typically marked by numerous visible mucosal nodules, generally measuring up to 0.5 cm in diameter, occasionally slightly larger [2].

In this report, we describe a case that mimicked acute appendicitis. Therefore, it is crucial for clinicians to be familiar with the disease’s clinical features to enable a structured evaluation and ensure appropriate perioperative management.

## Case presentation

A 29-year-old female patient presented with complaints of lower abdominal pain for the past six days. The pain was initially present around the umbilicus, which then shifted to the right side of the lower abdomen. It was sudden in onset, dull aching in nature, and gradually increased in severity. The pain was associated with intermittent fever, was low grade, and not associated with chills and rigor. The patient also had two episodes of non-projectile, non-blood-tinged, and non-bile-stained vomiting, and contained digested food particles.

The patient gave a history of similar complaints eight months back when she was diagnosed with acute appendicitis and was conservatively managed at a private hospital with intravenous fluid, analgesics, and antibiotics. The patient was discharged after five days, with no complications. The patient had no comorbid conditions nor a previous history of surgery.

On examination, the patient was febrile. Her pulse rate was 98 bpm. The rest of the hemodynamic parameters were within the normal range. On palpation of the abdomen, the patient had tenderness in the right iliac fossa. There was no guarding or rigidity. The per-rectal examination was unremarkable.

The patient was admitted and started on intravenous fluids, analgesics, and antibiotics. Chest radiograph and abdominal radiograph were done; both were unremarkable. A comprehensive blood screening was done. Only the white blood cell count was found to be raised at 15,800/cu mm. The rest of the investigations were found to be within the normal range. An ultrasonography was done, which revealed an appendix visualized measuring 10 mm in diameter with the presence of surrounding fat stranding and minimal peri-appendiceal fluid. Features were suggestive of acute appendicitis.

The patient was initially treated with non-operative management, which included adequate hydration, analgesics, and antibiotics. The patient was discharged in five days without any complications and was called after six weeks for an interval laparoscopic appendectomy.

**Figure 1:**
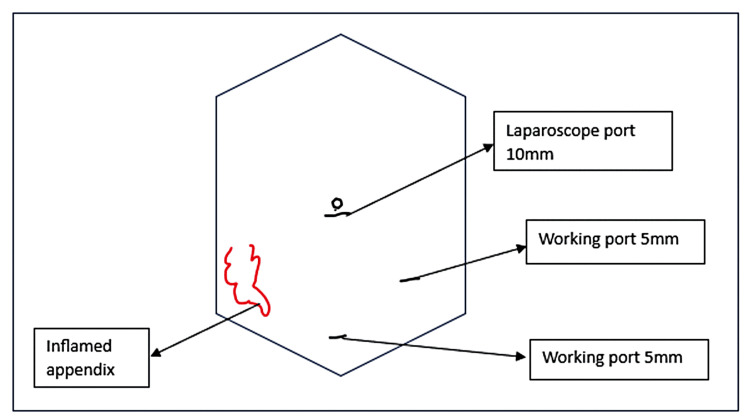
Port placement for laparoscopic appendectomy This figure is an original creation by the author.

After six weeks, the patient was taken for a laparoscopic appendectomy. Port placement was done as shown in Figure 1.

**Figure 2:**
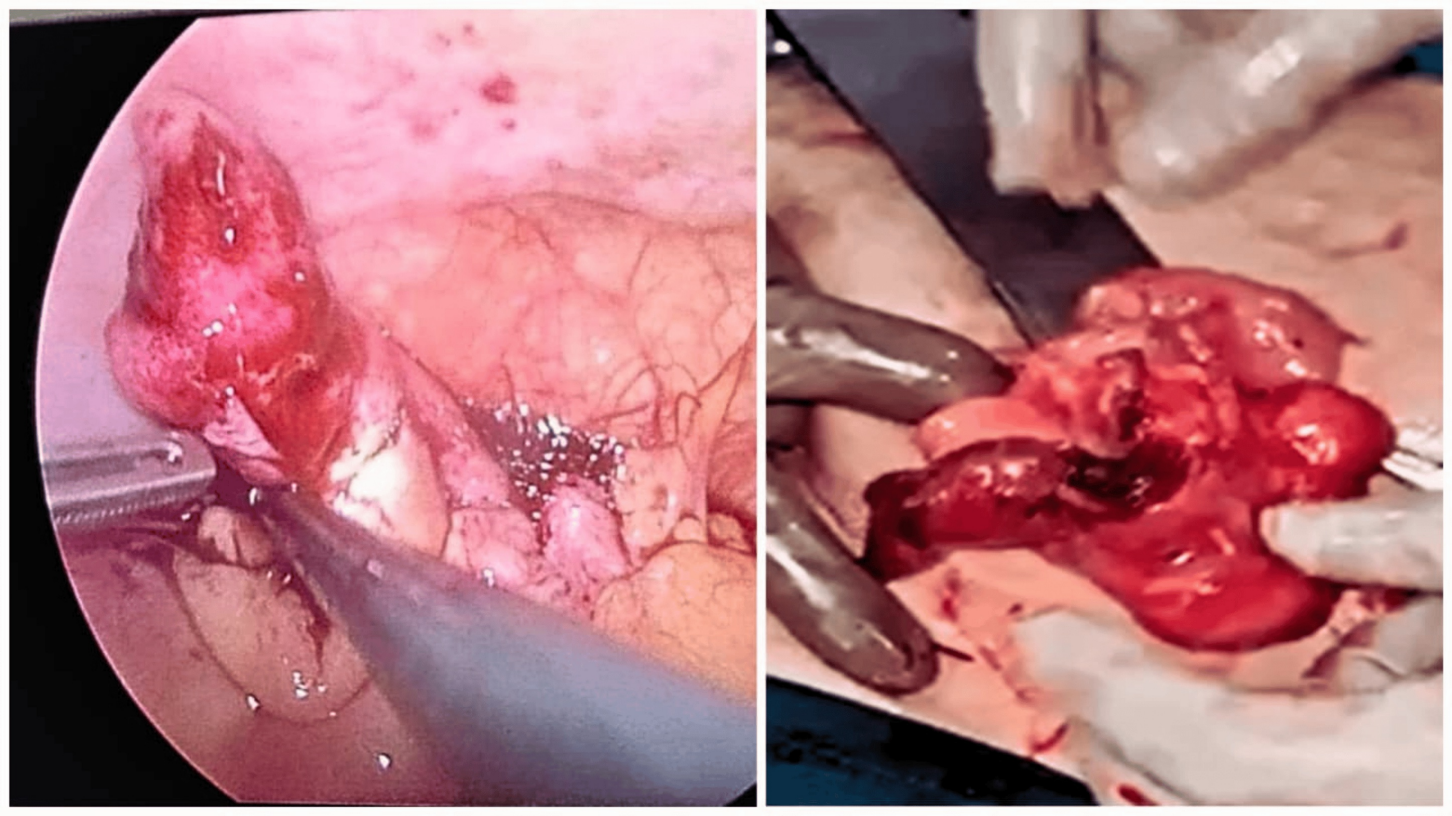
Intraoperative picture showing the thickened base of the appendix

Diagnostic laparoscopy was done, and the appendix was visualized. The base of the appendix was found to be thickened, as shown in Figure 2.

**Figure 3:**
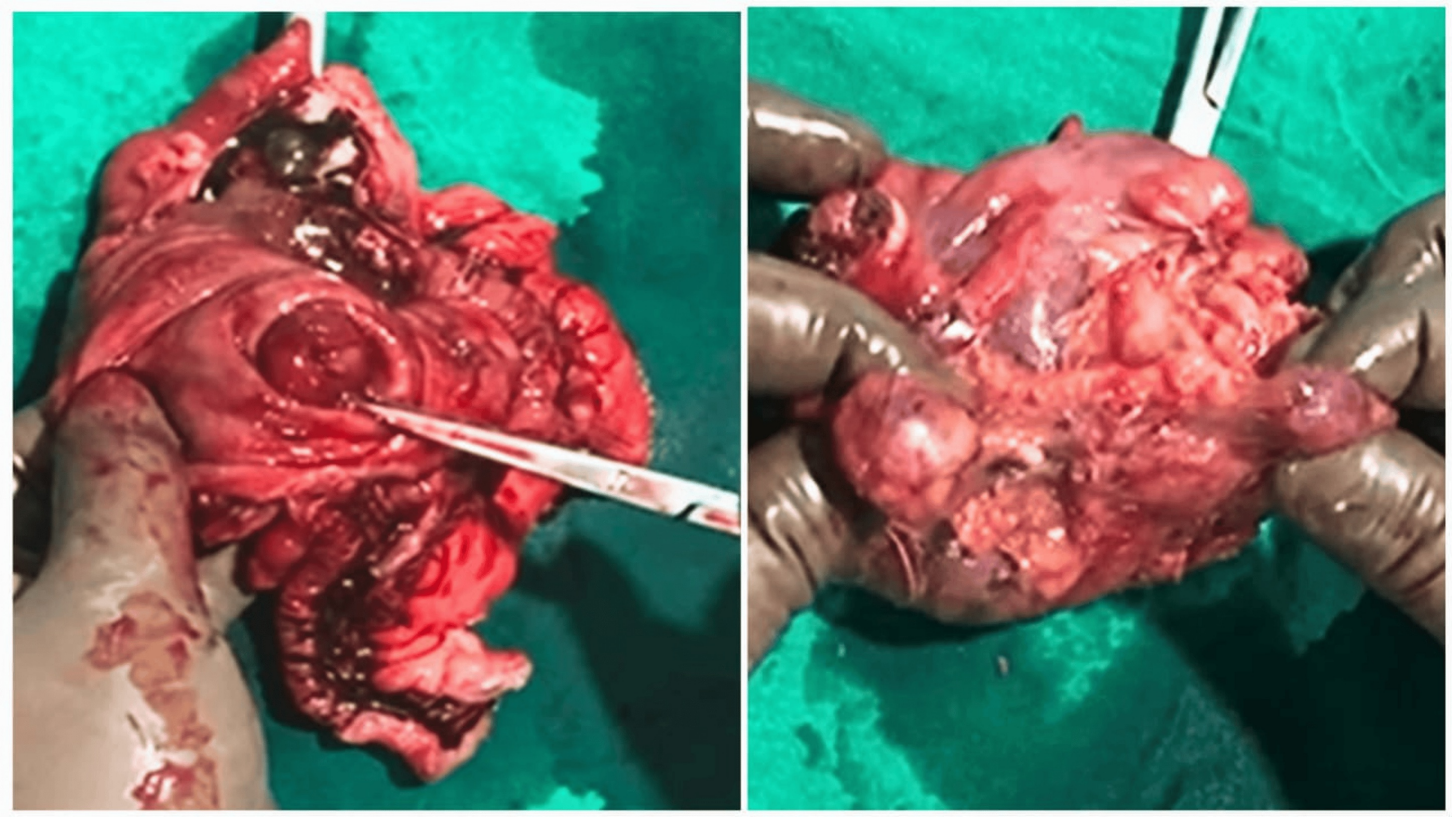
Cut section of the specimen showing a firm mass at the base of the appendix and multiple mesenteric lymph nodes

**Figure 4:**
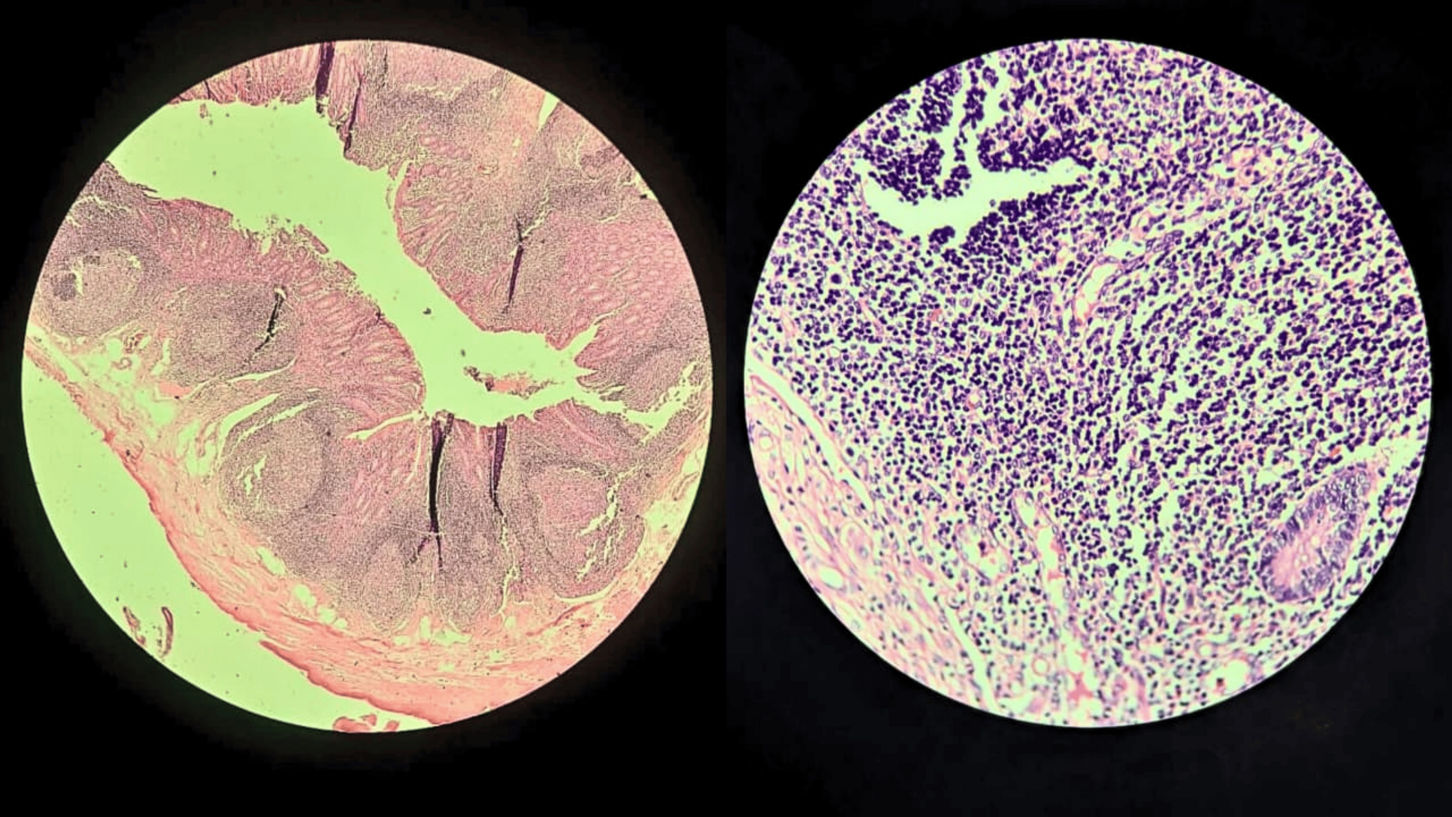
Histopathological examination of the specimen showed a markedly distended appendix with sheets and nodules of lymphoid cells in the submucosa (10x). Increased lymphocytic infiltration seen at 40x.

A decision was taken to do an exploratory laparotomy. The abdomen was opened with a midline incision. The appendix was delivered. A firm lump, approximately the size of 4*4 cm, could be palpated at the base of the appendix extending into the cecum. It was seen on further exploration that there were multiple, enlarged mesenteric and peri-appendiceal lymph nodes (Figure 3). A right hemicolectomy with an end-to-end ileo-colic anastomosis was performed, and the specimen was sent for histopathology (Figure 4).

Histopathological evaluation revealed a markedly distended appendix characterized by diffuse sheets and nodules of lymphoid cells, admixed with scattered plasma cells and neutrophils. Prominent sheets of macrophages were also identified. The lymphoid follicles were predominantly confined to the submucosa, with preservation of normal architectural patterns and no evidence of effacement. A total of 22 regional lymph nodes were examined, all of which were negative for metastatic disease. Collectively, these features were diagnostic of FLH.

The postoperative course was unremarkable. The patient was discharged one week following surgery and demonstrated complete clinical recovery at the one-month follow-up, with no evidence of complications.

## Discussion

FLH is a rare condition with an unknown etiology. Although lymphoid hyperplasia is frequently encountered in children and young adults, its florid variant in adults is extremely rare. It is a benign entity and is defined as an abnormal growth of secondary follicles primarily in the cortex of the lymph node, occurring without affecting the capsule, and is triggered by the activation of the B cell compartment [2].

FLH was first reported by Briquet in 1838 and was further researched radiologically by Marina Fiol and Rof-Carbello in 1941, and was given a new name called Enteritis follicularis [1]. Initially, it was believed that lymphoid hyperplasia contributed to the development of acute appendicitis by causing blockages in the appendix. However, this notion has largely been rejected. Chang analyzed over 3,000 appendectomy specimens and found that only 15 out of 1,711 acute appendicitis cases showed concurrent lymphoid hyperplasia. Moreover, 107 instances of lymphoid hyperplasia presented without any signs of acute inflammation [3]. This suggests that lymphoid hyperplasia is a physiological reaction to inflammation rather than a direct cause of appendicitis.

The site of involvement of the disease can be anywhere in the entire gastrointestinal tract, from the base of the tongue to the anus, with the most common being the terminal ileum and rectum.

The condition is thought to result from an antigenic response, such as Yersinia infection, Shigella, Campylobacter, and Adenovirus infection, triggering B-cell activation in the appendix's rich lymphoid network, most prominent in children and young adults. The hyperplasia typically reflects the immune system's response to these stimuli [1]. Adult florid variants remain idiopathic in many cases, including our case, with no identifiable trigger despite extensive workup. Recent studies link similar hyperplasias to post-viral responses (e.g., COVID-19 in colonic cases), suggesting immune dysregulation [4].

Patients of FLH often present with non-specific symptoms. They present with abdominal pain, often localized to the right lower quadrant. They can also present with nausea and vomiting. Occasionally, patients may present with a low-grade fever. These symptoms suggest acute appendicitis. Some patients may experience diarrhea or constipation, although these are less specific to FLH.

Patients are commonly evaluated using abdominal radiography and ultrasonography. On ultrasonography, findings may include nonspecific thickening or expansion of the hypoechoic lamina propria measuring greater than 0.8 mm, associated narrowing of the appendiceal lumen, and a noncompressible appendix with an increased maximal outer diameter of up to 6-8 mm. These sonographic features can closely mimic acute appendicitis, thereby posing a significant diagnostic challenge [5].

Computed tomography (CT) may demonstrate central luminal lucency with circumferential hyperattenuating wall thickening. In some cases, lymphoid tissue may appear as intraluminal fluid. Notably, periappendiceal fat stranding, commonly observed in acute appendicitis, is typically absent, which may aid in differentiation [5].

It can be very difficult to distinguish lymphoid hyperplasia of the appendix from acute appendicitis. As in our case, the patient was diagnosed with acute appendicitis on ultrasonography and was thus taken up for a laparoscopic appendectomy.

As it is a benign condition, nonoperative management should suffice for the palliation of symptoms. But it is often misdiagnosed as acute appendicitis, where appendectomy is the treatment of choice.

Our patient, who was diagnosed as a case of acute appendicitis, was conservatively managed and then planned for an interval appendectomy.

Laparoscopy showed thickening of the appendix. On exploration, multiple enlarged lymph nodes, approximately 22 in number, were found in the periappendicial mesentery. Thus, a decision was taken to do a right hemicolectomy. The patient was diagnosed with FLH on histopathology and was followed up for one month. She recovered well, with no complications

In effect, the definitive diagnosis of FLH can be established only through histopathological examination.

On gross examination, the appendix may appear grossly unremarkable, diffusely thickened, or, in some cases, exhibit a firm mass-like lesion at the base. A cut section typically reveals luminal narrowing secondary to submucosal proliferation of lymphoid tissue. Associated mesenteric lymph nodes may be enlarged, firm, and discrete [6].

Microscopic evaluation demonstrates marked hypertrophy of lymphoid follicles within the lamina propria and submucosa, characterized by numerous, well-formed germinal centers with prominent mitotic activity. Lymphoid aggregates are often arranged in clusters of 10 or more follicles, each measuring at least 2 mm in diameter. Expansion of these follicles results in attenuation of the submucosa and muscularis propria; however, there is no evidence of invasion or architectural effacement. The overlying mucosa is typically preserved, without dysplasia or ulceration [1,2,6].

Immunohistochemical analysis demonstrates a polyclonal B-cell population, evidenced by a mixed kappa and lambda light-chain expression. The lymphoid follicles are CD20-positive, with appropriately distributed CD3-positive T-cell zones. Germinal centers are negative for Bcl-2 and show a high proliferative index on Ki-67 staining, confined predominantly to the germinal centers. These findings support a reactive process and effectively exclude mucosa-associated lymphoid tissue (MALT) lymphoma and follicular lymphoma, which typically demonstrate monoclonality and aberrant Bcl-2 expression. Flow cytometric analysis further confirms B-cell polyclonality, and molecular studies using polymerase chain reaction show no evidence of clonal immunoglobulin gene rearrangement, thereby reinforcing the diagnosis of FLH [7]. Definitive diagnosis requires histopathology post-resection [8].

Rubin et al. noted that terminal ileal lymphoid hyperplasia can be categorized into two forms: common in childhood and rare in adults. The adult form is challenging to differentiate from low-grade lymphoma, with the key distinguishing factor being the absence of light chain restriction. In our case, we observed both kappa and lambda light chains through immunohistochemical staining. While some case reports have linked this condition to other systemic diseases, such as multiple intestinal polyposis, Gardner syndrome, and malignant lymphoma, these associations have primarily been documented in children under 10 years old.

Throughout the twentieth century, isolated case reports and small case series expanded knowledge of the condition. Rubin and Isaacson, in 1990, highlighted the diagnostic dilemma in adults, noting that FLH of the terminal ileum and appendix can closely resemble low-grade malignant lymphoma, both grossly and microscopically [6].

More recent case reports and reviews have emphasized its rarity in adults, its radiological mimicry of appendicitis and ileocecal malignancy, and its generally benign course [1]. Despite advances in imaging and pathology, FLH remains a diagnostic challenge, frequently discovered only after surgical resection for presumed appendicitis or carcinoma.

The potential for malignancy in adults remains uncertain, and our case did not exhibit any signs of cancer [1]. Further large-scale studies are needed to clarify the etiology and malignancy risk associated with this condition. Additionally, it can be mistakenly diagnosed as acute appendicitis or Crohn’s disease during surgery, as occurred in our case.

The differential diagnosis of FLH includes several important clinical and pathological entities. The most frequent mimic is acute appendicitis, as both conditions present with right lower quadrant pain, leukocytosis, and a thickened appendix on imaging. However, appendicitis typically demonstrates periappendiceal fat stranding and suppuration, whereas FLH lacks these changes and shows only follicular hyperplasia with preserved mucosal architecture [3]. Another critical consideration is low-grade non-Hodgkin’s lymphoma, particularly MALT lymphoma or follicular lymphoma. As described by Kumar V, Abbas AK, and Aster JC in Robbins and Cotran Pathologic Basis of Disease, although both entities can present with nodular lymphoid proliferation, FLH is characterized by preserved nodal architecture with well-formed germinal centers and polyclonal immunohistochemical findings, in contrast to lymphoma, which demonstrates architectural effacement and monoclonality [9]. Crohn’s disease may also resemble FLH due to terminal ileal involvement and prominent lymphoid aggregates, but it is differentiated histologically by transmural chronic inflammation, fissuring ulcers, granulomas, and mucosal architectural distortion, which are absent in FLH, as quoted by Briquet in Cruveilher’s Atlas of Pathological Anatomy. Vol II. Paris: Baillière; 1835-1842. Infectious ileitis, including Yersinia, Shigella, or adenovirus-related disease, is another potential mimic, producing mesenteric lymphadenopathy and ileocecal thickening. Unlike FLH, infectious ileitis demonstrates acute mucosal inflammation, necrosis, and identifiable microbial etiology [1,6]. Rarely, neoplastic conditions, such as carcinoid tumor or metastatic adenocarcinoma, may present with a mass lesion at the appendiceal base, but histopathology confirms their malignant nature [10]. Thus, careful integration of clinical, radiological, and especially histopathological findings is crucial for distinguishing FLH from these differentials.

FLH is generally regarded as a benign and self-limiting condition with an excellent prognosis [1,2]. In most cases, symptoms resolve either spontaneously or following surgical excision performed for suspected appendicitis or mass lesions. Most reported adult cases, including ours, showed uneventful postoperative recovery and no recurrence on follow-up, highlighting the favorable outcome of this entity.

Long-term follow-up may be warranted in some cases to monitor for any potential complications or associated conditions, although the risk is generally low [1].

## Conclusions

Florid lymphoid hyperplasia of the appendix is a rare entity that presents significant preoperative diagnostic challenges, as its clinical and radiologic features can closely mimic other abdominal pathologies, including acute appendicitis, inflammatory bowel disease, and small bowel neoplasms. A high index of clinical suspicion is therefore essential. Although imaging may contribute to preoperative assessment, definitive diagnosis is most often established only on histopathological examination. Heightened clinician awareness of this entity is crucial, as its recognition can directly influence diagnostic algorithms, prevent misclassification as malignant or inflammatory disease, reduce unnecessary surgical or extensive operative interventions, and improve patient counseling and management by ensuring its inclusion in the clinician’s armamentarium of differential diagnoses.
